# A comparison of four scoring methods based on the parent-rated Strengths and Difficulties Questionnaire as used in the Dutch preventive child health care system

**DOI:** 10.1186/1471-2458-8-106

**Published:** 2008-04-04

**Authors:** Mathilde R Crone, Anton GC Vogels, Femke Hoekstra, Philip DA Treffers, Sijmen A Reijneveld

**Affiliations:** 1 TNO Quality of Life, Leiden, The Netherlands; 2 Health Service Amsterdam, Amsterdam, The Netherlands; 3 Department of Child and Adolescent Psychiatry, Curium, Leiden University Medical Center, Oegstgeest, The Netherlands; 4 Department of Health Sciences/NCH, University Medical Center Groningen, Groningen, The Netherlands

## Abstract

**Background:**

Validated questionnaires can support the identification of psychosocial problems by the Preventive Child Health Care (PCH) system. This study assesses the validity and added value of four scoring methods used with the Strengths and Difficulties Questionnaire (SDQ) for the identification of psychosocial problems among children aged 7–12 by the PCH.

**Methods:**

We included 711 (of 814) children (response: 87%) aged 7–12 undergoing routine health assessments in nine PCH services across the Netherlands. Child health professionals interviewed and examined children and parents. Prior to the interview, parents completed the SDQ and the Child Behaviour Checklist (CBCL), which were not shown to the professionals. The CBCL and data about the child's current treatment status were used as criteria for the validity of the SDQ. We used four SDQ scoring approaches: an elevated SDQ Total Difficulties Score (TDS), parent-defined difficulties, an elevated score for emotional symptoms, conduct problems or hyperactivity in combination with a high impairment score, and a combined score: an elevated score for any of these three methods.

**Results:**

The Cohen's Kappa ranged from 0.33 to 0.64 for the four scoring methods with the CBCL scores and treatment status, generally indicating a moderate to good agreement. All four methods added significantly to the identification of problems by the PCH. Classification based on the TDS yielded results similar to more complicated methods.

**Conclusion:**

The SDQ is a valid tool for the identification of psychosocial problems by PCH. As a first step, the use of a simple classification based on the SDQ TDS is recommended.

## Background

Psychosocial problems such as behavioural, emotional, and educational problems are very prevalent among children and adolescents, and may interfere severely with everyday functioning. Only a minority of the children with such problems receive mental health care. In a study of more than 2,000 Dutch children, only 13% of the children with behavioural and emotional problems had been referred to mental health services in the year prior to the assessment [1]. Early treatment, however, may reduce these problems if they are accurately identified [2].

In the Netherlands, the Preventive Child Health Care (PCH) system is one of the most important low-threshold services for the early identification of emotional and behavioural problems in children. Physicians and nurses working in the PCH routinely offer preventive health care to all children aged 0–19 living in the Netherlands. More than 90% of all children undergo three to four assessments by a child health doctor or nurse during their school careers, in both primary and secondary school [3,4]. In the Netherlands, municipalities are obliged by law to guarantee proper access to this type of care free of charge, including the early identification of psychosocial problems.

However, several studies have shown that, when the PCH does not use validated questionnaires, only half of the children with emotional or behavioural problems are identified [3-5]. Validated questionnaires may help in the identification of these problems by the PCH [6,7]. For children aged 7–12 years, however, there is no short validated questionnaire for use by the PCH.

The Strengths and Difficulties Questionnaire (SDQ) is a promising option in this respect. It was developed by Robert Goodman to support the early identification of behavioural and emotional problems [8]. It is a brief measure covering the most important current domains of child psychopathology (i.e. emotional symptoms, conduct problems, hyperactivity-inattention, and peer problems) that can be completed by parents, teachers and the young people themselves. The SDQ Parent Form we used in our study consists of 25 symptom items, one item relating to the severity of problems as perceived by parents, and seven items assessing difficulties in functioning associated with the reported problems. The psychometric properties and validity of SDQ have already been shown to be good in a number of countries [9-13], including the Netherlands [14,15]. However, its appropriateness and added value for use by the PCH have not yet been assessed.

Bourdon *et al*. [16] used four SDQ scoring methods in a US setting to identify children who may have serious mental health difficulties. Their approach was based on the three components of the SDQ (symptom items, severity as perceived by the parents and impairment in functioning (see Method section for details)). The percentages of children identified varied according to the scoring method. Using service contact/use for a mental health reason as validation criterion, they found highly significant associations between service contact/use and each scoring method. The scoring method using parent-defined difficulties (severity perceived by the parents) identified the highest percentage of children with a service contact/use. Bourdon *et al*. [16] therefore concluded that parental judgement of the severity of children's difficulties may be a key indicator in bringing those difficulties to the attention of general medical and mental health professionals.

Contact with and use of mental health services is, in our view, of limited value as a measure for the validation of questionnaires such as the SDQ. Research has shown that many children with serious problems are not referred to such services [1]. If this variable is used as the main criterion, the children with problems who have no contact with mental health services will not be identified. Bourdon *et al*. did not have data relating to a validated overall instrument for emotional and behavioural problems, such as the Child Behaviour Checklist (CBCL), to validate the scoring methods. This study assesses the validity and added value of the four classification methods of the SDQ for the detection of emotional and behavioural problems by the PCH, using both the CBCL and current treatment for psychosocial problems as criteria for validity.

## Methods

### Population

We obtained our sample using a two-step procedure. In the first step, we selected a sample of PCH services. These services then collected data relating to children aged 7–12 years. Thirty-five child health professionals from nine PCH services participated in this study. A total of 814 parents and their children were asked to participate in this study: 10% refused to participate and 3% did not return the questionnaire, resulting in a response of 711 children (87%). Respondents were representative of the total sample in terms of age and gender, but non-response was higher for children of immigrant/minority origin (27.4% compared with 12.2% for children from Dutch origin).

### Measures and procedures

Data were obtained during routine health assessments. Before the assessments, parents filled out the CBCL and the SDQ. The parents gave both questionnaires to the child health professional, who passed them on to the researchers without opening them. The child health professionals interviewed the children and parents about mental health and background, and examined the children with the help of a structured questionnaire including questions on life events and current treatment for psychosocial problems. After each assessment, the health professional answered the following questions: 'Is the child currently being treated for psychosocial problems', 'Does the child have a psychosocial problem at present?' (yes, no), and scored the severity (mild, moderate or severe) and type of problem(s) identified using a pre-coded list.

The CBCL was used as a gold standard for parent reports about children's behavioural and emotional problems during the preceding six months [17]. The reliability and validity of the CBCL established by Achenbach were confirmed for the Dutch translation [17,18]. The CBCL consists of 20 competence items and 120 problem items. We used only the problem items. Parents indicated the presence of problems, choosing from one of three categories (no problem, sometimes/a little, often/a lot). We computed two broadband groups of syndromes – internalising and externalising – and a total problems score. Cases were subsequently allocated to a normal or a clinical range in accordance with the scoring distributions in the Dutch normative sample [18].

In this study, we used the parent version of the SDQ 4–16 [8,14,15]. The questionnaire consists of 25 symptom items describing positive and negative attributes of children and adolescents that can be allocated to 5 subscales of 5 items each: emotional symptoms, conduct problems, hyperactivity-inattention, peer problems, and pro-social behaviour. Each item has to be scored on a 3-point scale (0 = 'not true', 1 = 'somewhat true', and 2 = 'certainly true'). A total SDQ Total Difficulties Score (TDS) can be calculated by aggregating the scores for the emotional symptoms, conduct problems, hyperactivity-inattention, and peer problems subscales (range 0–40). The SDQ also contains an impact supplement that asks the parents about the severity of the problems as perceived by the parents and enquires about duration, distress, social impairment, and burden for others. A three-point scale is used for each item: 0 = not all all/only a little, 1 = quite a lot, 2 = a great deal. An impairment score was calculated by aggregating the scores for distress and social impairment [14,15].

The SDQ TDS and the SDQ subscales correlated significantly with the CBCL scores. The highest correlation coefficient was found between the CBCL total problem score and the SDQ TDS (r = 0.77) and the lowest correlation coefficient between the CBCL internalising problem score and the SDQ hyperactivity scale (r = 0.28).

We dichotomised the CBCL and SDQ for the analyses. For the CBCL, we used the standard Dutch cut-off points for dichotomising [18]. Dutch children tend to score lower on the SDQ than UK children; in the Netherlands about 6% of all children score above the UK cut-off point (≥ 17). Using this cut-off point would have led to low sensitivity indices (0.52 for a clinical CBCL score and 0.27 for 'currently being treated'). We therefore also computed sensitivity and specificity at a cut-off point that yielded a prevalence rate similar to that in the UK (10%). The most appropriate cut-off was therefore a SDQ TDS of 14 and higher.

Bourdon *et al*. [16] developed four SDQ scoring methods to identify children who may have serious mental health difficulties. The methods were based on the three components of the SDQ (symptom items, severity as perceived by the parents and impairment in functioning). Bourdon *et al*. classified children as having problems in four ways:

1. children with a score on the SDQ TDS above the cut-off point;

2. children whose parents perceived definite or severe difficulties on the impact supplement of the SDQ;

3. children with scores above the UK cut-off point for emotional symptoms, conduct problems, or hyperactivity-inattention in combination with an impairment score above the cut-off point;

4. combination: children classified as having problems using any of the first three methods.

These four classification methods were included in the analyses.

Child and family background characteristics assessed by the PCH were: gender, age, ethnicity, family characteristics (number of parents), income, educational level of the mother and employment status of the parent(s). Ethnicity was based on the native country of both biological parents. The country was coded as non-industrialised if at least one parent was born outside a member country of the Organisation for Economic Co-operation and Development or in Turkey.

### Analysis

The analysis assessed the validity of the four scoring methods and their added value for the identification of children with problems by the PCH. The validity of the different scoring methods was assessed using sensitivity and specificity indices, for 'currently being treated for psychosocial problems' and the dichotomised (normal/clinical) CBCL Total Problem, Internalising and Externalising scores as criteria. We will present the Cohen's Kappa to measure the agreement between the three scoring methods and the criteria 'currently being treated' and a clinical CBCL score.

We then determined the added value of the four classification methods, i.e. we assessed to what extent each of the four methods contribute to the distinction between children with and without a clinical CBCL score or treatment, after taking into account the identification by PCH based on clinical judgement after the standard health examination. To this end, we performed a stepwise logistic regression analysis with each of the criterion measures as the dependent variable. In the first step we included the identification by PCH in the analyses and in the second step we added the SDQ-based scoring methods. The significance of the change in the log likelihood ratio in the second step of the models was used to determine whether adding the classification methods contributed to a better distinction.

## Results

The mean age of the sample was 9.65 years. The other characteristics of the response group are presented in Table 1.

**Table 1:** Social and demographic indicators and differences between children with and without problems identified by four scoring methods

		SDQ TDS $	Parent-defined difficulties	High sub- scale/impairment	Combination of these three scores $
**Total (n = 707)**	*% (n)*	*%*	%	%	%

Elevated score		9.8	8.2	6.4	13.6
**Gender child**		**ns**	*	**ns**	**
Boy	49 (345)	11.9	10.7	7.5	17.7
Girl	51 (362)	7.7	5.8	5.2	9.7
**Age of the child#**		**ns**	**ns**	**ns**	**Ns**
7 years	11 (75)	4.0	1.3	1.3	5.3
8 years	7 (53)	11.3	15.1	5.7	20.8
9 years	28 (198)	10.6	9.6	7.1	14.1
10 years	22 (159)	10.1	8.2	5.7	14.5
11 years	23 (161)	10.6	6.2	7.5	12.4
12 years	9 (61)	9.8	11.5	9.8	16.4
**Ethnic background**		**ns**	**ns**	**ns**	**ns**
Netherlands or other industrialised country	83 (589)	9.2	8.0	6.6	13.2
Non-industrialised country	7 (46)	15.2	10.9	6.5	17.4
Unknown	10 (72)	11.1	8.3	4.2	13.9
**Family situation**		**ns**	**ns**	**ns**	**nS**
Two parents	86 (607)	9.1	8.1	5.6	13.0
One parent	10 (73)	16.4	9.6	12.3	19.2
Unknown	3 (27)	11.1	11.1	11.1	16.7
**Parenting situation**		**ns**	*	*	**ns**
2 biological parents	81 (574)	8.7	7.1	5.2	12.2
1 biological parent	14 (99)	16.2	13.1	11.1	21.2
Other/unknown	5 (34)	8.8	11.8	11.8	14.7
**Work situation**		*	**ns**	**	**ns**
No job	3 (22)	13.6	13.6	13.6	13.6
One full-time job	30 (212)	8.0	9.0	3.8	12.3
One part-time job	4 (27)	7.4	0.0	3.7	7.4
One full-time + one part-time job	42 (298)	9.1	7.0	6.4	12.8
Both part-time	8 (60)	5.0	10.0	3.3	13.3
Both full-time	3 (18)	11.1	5.6	0.0	11.1
Unknown	10 (70)	21.4	11.4	17.1	24.3
**Education**		*	**ns**	**ns**	*
(None) elementary school	3 (19)	21.1	15.8	0.0	31.6
Lower education	25 (178)	12.9	8.4	7.3	16.9
Medium education	32 (228)	10.1	8.8	5.7	13.6
Higher education	33 (236)	5.5	6.8	5.9	9.3
Unknown	6 (46)	13.0	8.7	10.9	15.2

* Chi Square: = p < 0.05; ** = p < 0.01; *** = p < 0.001.

$ Using an adapted Clinical SDQ cut-off; ≥ 14.

# The categorical variable was used: there were also no significant differences in mean age.

ns Not significant

### Validity

As indicated before, ten percent of the children scored above the adapted cut-off point. Eight percent had a parent-defined definite or severe difficulties score on the impact supplement of the SDQ and six percent had a high score for emotional symptoms, conduct problems or hyperactivity in combination with a high impairment score. Thirteen percent were classified as having problems identified by any of the three other classification methods. The Cohen's Kappa coefficient measuring the agreement between the three scoring methods varied from 0.49 to 0.59, which means a moderated agreement (0 is no agreement and 1 is perfect agreement). Four percent of the children had an elevated score on all three scoring methods.

Eight percent of the children had a clinical CBCL total problems score, 10% a clinical CBCL internalising score, 6% a clinical externalising score, and 7% were being treated for psychosocial problems. Table 2 presents the Cohen's Kappa, sensitivity and specificity indices for each of the four scoring methods. Kappa values ranged from 0.33 to 0.64 for the CBCL criteria and from 0.32 to 0.37 for the treatment status criterion. For each criterion, the 95% confidence intervals of the Kappa overlap. So the overall agreement of the four scoring methods with the criteria does not differ significantly.

**Table 2:** Test characteristics of the SDQ using clinical CBCL scores and treatment status as criterion; Area under Curve (AUC), Sensitivity, Specificity and 95% Confidence Intervals (95% CI), in relation to four scoring methods (n = 707).

	Elevated SDQ TDS*			Parent-defined difficulties			High sub-scale score/impairment			Combination of these three scores*		
	Kappa 95% CI	Sens. 95% CI	Spec. 95% CI	Kappa 95% CI	Sens. 95% CI	Spec. 95% CI	Kappa 95% CI	Sens. 95% CI	Spec. 95% CI	Kappa 95% CI	Sens. 95% CI	Spec. 95% CI

CBCL Total problems	0.64 (0.54–0.74)	0.75 (0.63–0.86)	0.96 (0.94–0.97)	0.45 (0.33–0.57)	0.50^a ^(.37–0.63)	0.95 (0.94–0.97)	0.54 (0.42–0.66)	0.52 (0.39–0.65)	0.97 (0.96–0.99)	0.55 (0.45–0.65)	0.80^a ^(0.70–0.91)	0.92 (0.90–0.94)
CBCL Internalising problems	0.47 (0.36–0.58)	0.51 (0.40–0.63)	0.95 (0.93–0.97)	0.46 (0.35–0.57)	0.46 (0.34–0.57)	0.96 (0.95–0.98)	0.43 (0.32–0.55)	0.39^b ^(0.28–0.50)	0.97 (0.96–0.99)	0.50 (0.40–0.60)	0.65^b ^(0.54–0.76)	0.92 (0.90–0.94)
CBCL Externalising problems	0.50 (0.38–0.61)	0.73 (0.59–0.86)	0.94 (0.92–0.96)	0.37 (0.24–0.49)	0.50 (0.35–0.65)	0.94 (0.93–0.96)	0.46 (0.33–0.60)	0.53 (0.37–0.68)	0.96 (0.95–0.98)	0.39 (0.29–0.50)	0.75 (0.62–0.88)	0.90 (0.88–0.92)
Treatment status	0.32 (0.20–0.44)	0.46 (0.32–0.60)	0.93 (0.91–0.95)	0.37 (0.24–0.49)	0.46 (0.32–0.60)	0.94 (0.93–0.96)	0.32 (0.19–0.45)	0.35 (0.22–0.49)	0.96 (0.94–0.97)	0.34 (0.24–0.45)	0.60 (0.46–0.74)	0.90 (0.87–0.92)

*Using an adapted Clinical SDQ cut-off; ≥ 14.

^a ^Sensitivity is significantly lower with a scoring method based on parent-defined difficulties than with a scoring method based on the combination score.

^b ^Sensitivity is significantly lower with a scoring method based on high subscale score and impairment than with a scoring method based on the combination score

The highest sensitivity for the identification of a clinical CBCL total problems score was found for the combination score (0.80). The combination score was also most sensitive for a clinical CBCL internalising score and externalising score. However, this score had the lowest specificity (varying from 0.90 to 0.92). Almost all 95% confidence intervals for sensitivity overlapped, meaning that these differences in sensitivity are not significant. We found two significant differences in sensitivity (based on non-overlapping confidence intervals): compared to parent-defined problems, the combination method is more sensitive to a clinical CBCL total problem score; compared to a high-subscale and impairment score, the combination method is more sensitive to a clinical CBCL internalising score.

### Added value

In the first step of these analyses we assessed the likelihood (odds ratio (OR)) of a clinical score on the CBCL scales or of 'currently being treated' if the health professional identified psychosocial problems. Children who were identified by the health professional as having psychosocial problems had a significant higher odds of having a clinical score on the CBCL scales or of 'currently being treated'; CBCL total, OR = 6.81 (3.78–12.28); CBCL internalising, OR = 6.35 (3.78–10.68); CBCL externalising, OR = 5.61 (2.86–10.90); Currently being treated, OR = 10.93 (5.45–21.95)). In the next step we assessed the added value of the four scoring methods to the identification by the health professional. Table 3 presents the odds of having a clinical score on the CBCL scales and of 'being currently treated' if a child was classified as having problems by one of the scoring methods. The Odds Ratios presented are adjusted Odds Ratios, taking into account the identification of problems (yes./no) by the child health professional. In other words, the table indicates to which degree a specific SDQ scoring method increased the likelihood of identification of children with a clinical CBCL score or 'currently being treated' compared with only including the assessment by the child health professional.

**Table 3:** Results from four separate logistic regression analyses for each scoring method on clinical CBCL scores and treatment status, taking the identification by the PCH into account (n = 707); adjusted odds ratios (OR) and 95% Confidence Intervals (95% CI), for identification by PCH and the SDQ scoring method

	Clinical CBCL total clinical score	Clinical CBCL Internalising problems score	Clinical CBCL Externalising problems score	Being treated for psychosocial problems
	Adjusted OR (95% CI)	Adjusted OR (95% CI)	Adjusted OR (95% CI)	Adjusted OR (95% CI)

Elevated SDQ TDS cut-off ≥ 14				
PCH detects problems yes (vs no)*	2.59 (1.23–5.47)	3.58 (2.01–6.40)	1.92 (0.85–4.32) ns	7.20 (3.46–14.99)
Elevated SDQ score yes (vs no)	49.93 (23.67–105.36)	12.92 (6.97–23.92)	31.69 (13.96–71.98)	5.54 (2.78–11.06)
Parent-defined difficulties				
PCH detects problems yes (vs no)*	3.57 (1.85–6.89)	3.56 (1.99–6.35)	2.79 (1.30–5.97)	6.72 (3.20–14.11)
Elevated SDQ score yes (vs no)	16.33 (6.20–24.45)	12.52 (6.52–24.03)	10.44 (4.85–22.48)	7.02 (3.44–14.34)
High sub-scale score/impairment				
PCH detects problems yes (vs no)*	3.47 (1.76–6.85)	3.96 (1.80–6.29)	2.55 (1.17–5.57)	7.72 (3.72–16.00)
Elevated SDQ score yes (vs no)	26.76 (12.53–57.17)	13.79 (6.74–28.21)	19.64 (8.81–43.81)	5.68 (2.66–12.11)
Combination of these three scores$				
PCH detects problems yes (vs no)*	1.96 (0.95–4.06) ns	2.63 (1.43–4.85)	1.73 (0.78–3.83) ns	5.75 (2.70–12.25)
Elevated SDQ score yes (vs no)	36.04 (16.66–77.96)	15.17 (8.26–27.88)	21.29 (9.24–49.06)	6.75 (3.40–13.41)

* adjusted odds ratios: adjusted for the scoring method.

$ Using the adapted Clinical SDQ cut-off; ≥ 14.

Adding any of the SDQ scoring methods into the equation always led to a significant change in the log likelihood ratio. The ORs for all the scoring methods were significant, regardless of the criterion used. Overall, the SDQ improves the identification of children with an elevated CBCL internalising problems score less well than the identification of children with an elevated clinical CBCL total problems and externalising problems score. An elevated SDQ TDS had most added value for the prediction of a clinical CBCL compared to the other three classification methods. The combination score and the parent-reported difficulties added most to the prediction of 'currently being treated'. However, once again, the 95% confidence intervals of the ORs overlapped for all criteria, meaning that there were no significant differences between the scoring methods.

## Discussion

This study assessed the suitability of the SDQ for the early detection of psychosocial problems among children aged 7–12 years by the PCH. We looked at the validity of four SDQ-based scoring methods: 1) the SDQ TDS, 2) the definite or severe difficulties perceived by parents using the impact supplement of the SDQ, 3) an elevated score for emotional symptoms, conduct problems, hyperactivity in combination with an elevated impairment score and 4) a combination method: an elevated score for any of these three methods. The results show that all four scoring methods of the SDQ are valid and have added value for the identification of psychosocial problems among children. We found that the SDQ TDS and the combination method (which includes the elevated TDS) were most sensitive for elevated CBCL scores, and that the difficulties identified by parents and the combination method were most sensitive for children currently being treated for psychosocial problems. However, most differences in sensitivity between the scoring methods were not statistically significant. The exception was the combination method, which was significantly more sensitive for an elevated CBCL total problem score than the scoring method based on parent-defined difficulties. The combination method was also statistically more sensitive to an elevated CBCL internalising problems score compared to an elevated score for emotional symptoms, conduct problems or hyperactivity in combination with a high impairment score.

Finally, the SDQ TDS added most to the identification of psychosocial problems by the PCH, although the differences between the scoring methods were again not statistically significant.

Bourdon *et al*. [16] found significant differences between the scoring methods: an elevated SDQ TDS alone distinguished less well between children with and without service contact/use than parent-reported difficulties and an elevated score for emotional symptoms, conduct problems, or hyperactivity-inattention in combination with an elevated impairment score. By contrast, we found no significant differences in sensitivity or added value between the SDQ TDS and the other scoring methods. This may be due to the fact that our study sample was much smaller. The number of cases in our sample was therefore rather small and the power of tests for sensitivity and for the OR in the logistic regressions is therefore rather small.

The percentage of children scoring above the UK cut-off point in our study was lower than in the UK. In Germany and the United States the 10% cut-off point (≥ 16) also tended to be slightly lower than in the UK but it was much closer to the UK than the cut-off point in this study [13,16,19]. Another Dutch study, however, found the same 10% cut-off point of 14 for the parent SDQ as this study did [20]. The authors of the other Dutch study concluded that a possible reason for this lower cut-off was the substantial level of non-response among parents (response was 63%) [14,20]. In the present study, the response was much higher (87%) and the effect of non-response is therefore smaller. A study of the CBCL showed that Dutch parents also reported fewer problems on the CBCL than US parents but this did not apply to German parents, suggesting that it is a structural pattern [21]. We therefore believe that the lower SDQ scores in the Netherlands are not the result of some flaw in the study, but that they reflect a higher level of well-being among children in this sample (compare, for example UNICEF [22]).

The SDQ scores in this general population sample are most sensitive for a CBCL total problem score and least sensitive for internalising problems and current treatment. The impact supplement enhanced the identification of internalising problems slightly, but sensitivity remained lower than for the total problems score. This concurs with the findings of Goodman, who indicates that 'Not surprisingly, the algorithm seems most likely to miss children with relatively encapsulated symptoms that are not well covered by the SDQ'. It is important to mention that Goodman refers to a multi-informant algorithm (parent, teachers, and self-reports from older children) in which he found a greater likelihood of missing encapsulated or internalising problems. He proposes that 'if researchers or clinicians want to detect as many emotional or hyperactivity disorders as possible, they would be well advised to use the SDQ prediction for "any disorder" rather than for "emotional disorders" or "hyperactivity disorders". A second-stage screening procedure can then be used to detect which SDQ "positive" children have the disorder of particular interest' [[23], pages 537–538].

### Strengths and limitations

This study has important strengths but also some limitations. One strength is the high response rates. One limitation is however that the largest cities were not included in the sample, which means that the sample is not representative for the Dutch population; the percentage of children with a non-industrialised origin that participated in our study is therefore smaller than in the total population of children in this age group. The education level of the parents is also higher than in the national population. This could mean that the results of the present study are an underestimate and that children with psychosocial problems are not fully represented.

Another limitation is that the evaluation of questionnaires for emotional and behavioural problems is always hampered by difficulties in the choice of a gold standard: there is simply no definitive indicator of such problems. This study therefore adopted a common strategy to overcome this problem: in our study we included both the CBCL and current treatment as validation criteria. One of the problems, however, is that both the CBCL and the SDQ are completed by parents. This probably leads to a higher correlation between these two instruments because both instruments rely on the opinion of the parents. Clinical assessments, like psychiatric interviews, do not suffer from these problems and could therefore be more convincing as a criterion. However, we could not use psychiatric interviews in this study because of the costs and burden for the parents. Skovgaard *et al*. [24] also indicate that screening of a whole population can be conducted using an instrument such as the CBCL, and that diagnostic classification should take place in a second stage with a combination of psychometric and clinical approaches. These clinical assessments are expensive and time-consuming and should be restricted to smaller samples consisting of, for example, individuals identified by screening procedures such as the CBCL. In the Netherlands, the PCH is an important service for the identification of these high-risk children in the population as a whole. The CBCL is technically adequate for this first step in the identification of psychosocial problems, but it is too long, too time-consuming and therefore too costly, and not suitable for use in the PCH. The extent to which another, shorter, instrument can replicate the global classification of the CBCL will then be a valid measure of the suitability of this instrument.

At the same time, the inclusion of both treatment status and the CBCL as measures in our study is, in our view, a major advantage compared to the study of Bourdon *et al*. [16], since using contact with services as the only criterion neglects the fact that many children with serious problems never contact services because of their problems.

## Conclusion

The results of this study show that use of the SDQ can provide effective support for the PCH in the identification of psychosocial problems among children. The routine use of an instrument of this kind in the PCH is therefore recommended. For a first identification of children with problems by the Dutch PCH, only the use of the SDQ Total Difficulties Score is justified since more complicated and time-consuming scoring and classification methods do not significantly improve identification.

## What this paper adds

The SDQ as a short instrument for the detection of psychosocial problems among children can provide effective support for the identification of these problems in preventive child health care. After comparing four scoring methods of the SDQ, it can be concluded that for a first identification the use of only the SDQ Total Difficulties Score is justified.

## Competing interests

The author(s) declare that they have no competing interests.

## Authors' contributions

MRC contributed to the acquisition of data, the analysis and interpretation of data, and the drafting of this paper.

AGCV contributed to the conception and design, acquisition of data, and the analysis and interpretation of data. He was involved in revising the manuscript.

FH contributed to the analysis and interpretation of data.

PDAT contributed to the revision of the intellectual content of the paper.

SAR contributed to conception and design, and the analysis and interpretation of data, and was involved in revising the paper.

Finally, all authors read and approved the final manuscript.

## Pre-publication history

The pre-publication history for this paper can be accessed here:


